# Institutional Notes and News

**Published:** 1909-09-25

**Authors:** 


### Institutional Notes and News.

### A NEW INFIRMARY FOR PERTH.

It has now been decided to erect a new Infirmary for Perth city and county. The sum subscribed at present is only about £6,000 short of the estimated expense of £36,800. The building will be a modern, up-to-date institution, erected at Western Avenue, Glasgow Road. Mr. James Miller, architect, Glasgow, the architect of the Glasgow Royal Infirmary, to whom has been entrusted the planning of the new building, has drawn up specifications for a modern hospital, thoroughly up-to-date, with 104 ward beds, 8 special ward beds, and 8 other beds—in all, 120 beds—with accommodation for 32 nurses, 25 servants, out-patients' department, laundry, etc., at a total cost of £36,800. The amount received for reconstruction of the present buildings is £15,699 18s. 3d., and of this £9,167 13s. 3d. is available for a new infirmary. The special fund for the new infirmary amounts to £4,830 14s. 5d.; the subscription from the Glovers' Incorporation is £200; accumulation of interest, £801 1s. 4d.; legacies earmarked, £7,000; estimated price of old infirmary buildings, £10,000, leaving available for the erection and completion of a new infirmary £32,000. Only £6,000 remains unprovided for.

### THE NORWICH HOSPITAL.

His Majesty the King has signified his intention to lay the foundation stone of the first of the series of new buildings of the Norfolk and Norwich Hospital on the occasion of his visit to Norwich on October 25. The ceremony will take place after luncheon. His Majesty will be received at the hospital by the representatives of the institution, and will then be asked to lay the foundation stone of the building which is to form the new isolation block and the new septic block. His Majesty has himself subscribed 250 guineas towards the £50,000 which will be required, and the total subscription list stands to-day at £12,620, so that, in round figures, something like £37,000 is still needed to complete the extension. The need for the new isolation block and the new septic block is great. The only accommodation at the present time for cases of infectious disease consists of two rooms on the ground floor of the old wing of the hospital, at best a makeshift. The irregular wooden floors, with wide intervals between the boards, were laid about a hundred years ago; and the walls are such that it is impossible to wash them down. Cases of an infectious nature, such as scarlet fever, measles, whooping cough, and mumps, are bound to occur from time to time amongst the inmates of the hospital, and have occurred, and more than once in the recent history of the hospital outbreaks have taken place which would have been nipped in the bud had the proper facilities for isolation been possible.

### ROYAL VICTORIA INFIRMARY, NEWCASTLE.

At the last quarterly Court of Governors of this Infirmary, under the presidency of Sir George Hare Philipson, Chairman of the House Committee, a presentation was made to Sir Riley Lord in recognition of the valuable services he has rendered to the Infirmary. The gift took the form of an illuminated album containing an address, three water-colour sketches, and the signatures of the governors, enclosed in an oak casket. In making the presentation, the Chairman stated that it was a small acknowledgment of Sir Riley's valuable services as Chairman of the Queen's Commemoration Infirmary Fund, Chairman of the Building Committee of the new Infirmary, Chairman of the Subscriptions Committee, and in various other positions in connection with the committees of that institution; and in admiration of his action during his first mayoralty in commencing and in carrying to a successful issue the scheme for obtaining from the general public £100,000 as a commencement towards the buildings of the Infirmary.

The address was as follows:—

"The Governors of the Royal Victoria Infirmary, Newcastle-upon-Tyne, at their quarterly court held on May 27, 1909, in testimony of their admiration for Alderman Sir Riley Lord, J.P., and in recognition of the eminent services which he has rendered to the institution—and of such may be specially mentioned his inauguration, during his first mayoralty, 1895-1896, of the Queen's Commemoration New Infirmary Fund, whereby over one hundred thousand pounds was subscribed for the building of the new Infirmary—his valuable services as the Chairman of the Building Committee of the new Infirmary, and as the Chairman of the Subscriptions Committee, have pleasure in appointing him a President of the Royal Victoria Infirmary.

"Sir George Hare Philipson (Chairman).

"W. J. Sanderson (Vice-President)."

Sir Riley Lord, in returning thanks, remarked that the Infirmary was an institution which appealed to the hearts and consciences of all the people, whether they were rich or poor. He believed that, first of all, their success had been due to the love and affection the people had for Queen Victoria; they wished to do something to manifest their appreciation of her good sixty years' reign. Then, in the next place, everybody seemed to realise the need for a new hospital, the old one being utterly inadequate in every respect, except in regard to its position near the railway station. The new Infirmary cost £325,000, and, comparing the cost with the cost of other infirmaries built subsequently, the cost of the Newcastle Infirmary worked out at a less sum than that of other hospitals. A little over nine-tenths of the money raised for the building of the Infirmary came from persons who were never likely to be patients of the charity, and slightly less than one-tenth was obtained from workmen.

### THE CARDIFF INFIRMARY.

At the last ordinary monthly meeting of the board of management of Cardiff Infirmary, Colonel Bruce-Vaughan presiding, the Chairman announced the receipt of a communication from Sir W. T. Lewis to the effect that Mrs. John Nixon would lay the foundation-stone of the new wing on October 21, at three o'clock. The addition, costing £33,000, to which Mrs. Nixon has generously contributed 10,000 guineas, is in memory of her late husband, Mr. J. Nixon (Nixon's Navigation Colliery). The Chairman also announced that a grant of £1,500 had been made by the Treasury for foundation of a Cardiff medical school. The progress of the infirmary in the last few years from a building point of view, and the excellence of its staff, had contributed in no small degree to the allocation of the grant for a medical school. Dr. MacAlister (one of Sir Thomas Raleigh's committee), on his visit to Cardiff Infirmary last year, laid special stress on the provision for clinics in the new out-patient department, and the efficiency of every other department. The doctor's observations on the possibilities of Cardiff as a school of science had undoubtedly led to the recommendation by his committee of this grant. Probably the first step which would be made by the council of the college would be the appointment of a professor of pathology. They had made provision for this appointment by including in the new wing scheme the provision of buildings which would cost, including equipment, £4,600. The equipment in every department would be thoroughly up to date. The financial statement pointed to an overdraft of £21,160 19s. 11d.

### CLEVELAND CONVALESCENT HOME.

The Right Hon. Herbert Samuel, M.P. for Cleveland, visited Saltburn on Saturday for the purpose of performing the opening ceremony of the new Workmen's Convalescent Home for the Club and Institute Union. The building, which was formerly a school, originally cost £16,000, but was recently purchased by the union for £6,000, and the alterations cost £4,000. The exterior is of lofty and massive character. The interior, however, did not in its arrangements and fittings meet the growing demand which exists amongst workmen for a higher standard of comfort, and some £2,500 has been expended in removing the "dormitory" and "institution" appearance and in fitting it as a manor house or first-class hotel. A further expenditure of £2,000 has been necessary for furniture. The Home, which will accommodate 54 residents, will be supported entirely by the Union and its clubs, and no appeal is made for any outside help, following in this the practice initiated with the establishment of the Home at Pegwell Bay. The establishment of a third Home on the West Coast is under consideration, and a fourth in the South West also.

### NEW HOSPITAL AT KILMARNOCK.

The infectious diseases hospital, which the Corporation of Kilmarnock has erected at Kirklandside, was formally opened last week. The hospital consists of seven different blocks, and provides accommodation for forty-eight beds, a number of which will be available for patients from the neighbouring burghs of Galston, Newmilns, and Darvel. The total cost of buildings, furnishings, site, etc., amounts to about £20,000. The opening ceremony was performed by Mrs. Robertson, wife of Bailie Matthew Robertson, convener of the Public Health Committee, who was presented with a gold key from the architect, Mr. James Hay, and the contractors. A company numbering about three hundred ladies and gentlemen inspected the buildings, and afterwards were entertained to luncheon in one of the wards, Provost Gemmill presiding and Bailie Robertson officiating as croupier. Councillor W. F. Anderson, Glasgow, proposed "The Kilmarnock Burghs Infectious Diseases Hospital," stating that this was one of the most complete hospitals it had been his lot to see during his experience of eighteen years. Bailie Robertson replied. The architect afterwards presented the Provost and Bailie Robertson with a handsome rose bowl each as a souvenir of the occasion.

### HOSPITALS AND THE BUDGET.

An interesting discussion took place at a recent meeting of Arbroath Infirmary directors with regard to certain proposals contained in the Budget referring to death duties. The question was raised by Mr. W. K. Macdonald, the clerk, who said it appeared to him that the provision in the Budget making any gifts or charitable subscriptions made five years before a man's death liable to death duties might seriously affect the Infirmary, because while a man might be quite willing to give out of his income a charitable subscription he might not like that his heirs at his death should be called upon to pay duty on all of these sums. It was both a wrong and an unwise thing to penalise charitable subscriptions and make them liable to these death duties. These subscriptions were naturally paid out of a man's income, and if he paid income-tax on that it seemed a sort of double tax. One could quite understand death duties being charged if a man gave away part of his capital. It might be right enough to endeavour to frustrate any attempt to evade the death duties in that way, but the case was different as regards a subscription. If they put a shilling into the church plate that apparently would be liable to duty if the Government found out that they had given it. That seemed to be rather absurd.

In the discussion that followed the clerk said that in regard to the Infirmary the authorities could call for the production of the Infirmary accounts, and they would see everything that was given.

Mr. Alexander Balfour of Inchock thought it was an extraordinary thing in these days of freedom and liberty that a man who had been careful of his means and was trying to benefit others for the good of the community should have his money laid hold of—that if he chose in his lifetime to give away money to the Infirmary or to any friend or any person, that it should be taxed. Whatever their private opinions might be, he did not think they, as Infirmary directors, were at liberty to allow this to pass without protecting that valuable institution of theirs, which had done noble service for the town and district in bygone days. Mr. A. R. Duncan of Parkhill suggested that instead of making a regular petition they should point out that surely a mistake had been made in the Budget. The old clause was that if a man gave anything away in his lifetime to anyone else that the duty had to be paid if the transaction occurred within one year of his death. At that time there was no question of collecting death duties upon subscriptions, even although they were paid within one year before death, and he thought the new Act was simply meant to say that this one year shall be lengthened to five years, and that there could be no intention of taxing subscriptions in the way that had been stated. He thought the course to adopt was to draw up a sort of memorandum, to be sent to the Chancellor of the Exchequer, pointing out that if his Act were carried out in its entirety it would involve a payment of duty upon subscriptions.

It was finally agreed to remit the matter to the Finance Committee.

